# A Dissertation on the Causes and Effects of Disease, Considered in Reference to the Moral Constitution of Man

**Published:** 1838-07

**Authors:** 


Art. IX.—*A Dissertation on the Causes and Effects of Disease, considered in Reference to the Moral Constitution of Man.* By Henry Clark Barlow, m.d.—*Edinburgh,* 1837. 8vo. pp. 79.

This dissertation, which appears to have been written as an inaugural essay, presents a favorable example of the habits of mind generated in the Edinburgh school of medicine, in which the practical part of physic has long flourished in combination with such philosophical views as have found much less favour elsewhere. Dr. Henry Barlow has shown, with copious references to authorities and examples, that diseases arise, for the most part, from the neglect of moral and physical laws; and are not so much to be considered evils as remedies for evils. He has also laboured, not unsuccessfully, to show that the phenomena of disease are so regulated as to produce the intended end with as little inconvenience as could possibly be united with the attainment of such end; and that, even beyond the proposed object, certain results flow from them which are conducive to the benefit of mankind. Such a dissertation cannot but be viewed as a work of most favorable promise, exhibiting, as it does, marks of extensive reading, just reflection, and elevated views.

